# Altered Velocity Processing in Schizophrenia during Pursuit Eye Tracking

**DOI:** 10.1371/journal.pone.0038494

**Published:** 2012-06-05

**Authors:** Matthias Nagel, Andreas Sprenger, Susanne Steinlechner, Ferdinand Binkofski, Rebekka Lencer

**Affiliations:** 1 Department of Psychiatry and Psychotherapy, University of Luebeck, Luebeck, Germany; 2 Department of Neurology, University of Luebeck, Luebeck, Germany; 3 Neuroimage Nord, Department of Systems Neuroscience, University Clinic Hamburg Eppendorf, Hamburg, Germany; 4 Asklepios Klinik Nord – Wandsbek, Clinic of Psychiatry and Psychotherapy, Hamburg, Germany; 5 Department of Psychiatry and Psychotherapy, University of Muenster, Muenster, Germany; 6 Division for Cognitive Neurology, Department of Neurology, RWTH Aachen University, Aachen, Germany; University of Muenster, Germany

## Abstract

Smooth pursuit eye movements (SPEM) are needed to keep the retinal image of slowly moving objects within the fovea. Depending on the task, about 50%–80% of patients with schizophrenia have difficulties in maintaining SPEM. We designed a study that comprised different target velocities as well as testing for internal (extraretinal) guidance of SPEM in the absence of a visual target. We applied event-related fMRI by presenting four velocities (5, 10, 15, 20°/s) both with and without intervals of target blanking. 17 patients and 16 healthy participants were included. Eye movements were registered during scanning sessions. Statistical analysis included mixed ANOVAs and regression analyses of the target velocity on the Blood Oxygen Level Dependency (BOLD) signal. The main effect *group* and the interaction of *velocity*×*group* revealed reduced activation in V5 and putamen but increased activation of cerebellar regions in patients. Regression analysis showed that activation in supplementary eye field, putamen, and cerebellum was not correlated to target velocity in patients in contrast to controls. Furthermore, activation in V5 and in intraparietal sulcus (putative LIP) bilaterally was less strongly correlated to target velocity in patients than controls. Altered correlation of target velocity and neural activation in the cortical network supporting SPEM (V5, SEF, LIP, putamen) implies impaired transformation of the visual motion signal into an adequate motor command in patients. Cerebellar regions seem to be involved in compensatory mechanisms although cerebellar activity in patients was not related to target velocity.

## Introduction

Smooth pursuit eye movements (SPEM) are needed to keep slowly moving visual objects within the fovea. SPEMs are controlled by both retinal signals, i.e. the target’s retinal slip velocity, and extraretinal signals, i.e. internal representations of target and eye velocity. The maintenance of smooth pursuit is driven by a combination of retinal and extraretinal mechanisms, with their loadings depending on the extent of experience with the pattern of target motion and the predictability of the stimulus [1].

Up to 80% of patients with schizophrenia are impaired in maintaining SPEM velocity [2], [3] and about 50% of non-affected first degree relatives show similar deficits, suggesting a familiar or genetic nature [2], [4]. Despite the fact that pursuit abnormalities in patients have been shown to be stable over time and mostly independent of symptom state and, with some exceptions, independent of medication [5], patient’s SPEM performance highly depends on the task demands. We have recently shown that patient’s SPEM are unimpaired with stimuli that do not require constant dynamic adjustments of pursuit velocity and acceleration as with sinusoidal tasks, or abrupt reversals in target direction as with triangular waveforms [6]. Another study showed that pursuit of even unpredictably moving sinusoidal targets can be performed by patients as well as by healthy participants if maximum target speed is low [7]. This finding indicates target speed processing as a crucial factor for SPEM performance in patients with schizophrenia.

One cause for SPEM dysfunctions in patients may be alterations in visual motion processing as has been concluded from psychophysiological studies that showed reduced velocity discrimination in patients [8], [9]. Visual area V5 is regarded as the core region for motion processing receiving both retinal and extraretinal input. In a recent fMRI study we reported a reduced correlation between V5 activation during passive visual motion processing and activation of the anterior parietal sulcus during pursuit in patients [7]. This finding suggests that the utilization of the motion signal derived from V5 and its transfer to sensorimotor systems is altered in patients. In another fMRI-study, we found a less strong correlation between the measured pursuit eye velocity and the BOLD-signal (Blood Oxygen Level Dependency) in visual motion processing area V5 in patients compared to healthy participants [10]. It is unclear whether this finding reflects impaired retinal motion processing or rather altered extraretinal information processing [11], [12], [13], [14].

Extraretinal information processing can be evaluated by intervals of invisible targets that are interspersed during the target movement, and by instructing the subjects to follow the imagined target during the blanking interval, so that pursuit is driven by the predicted target trace from prior experience with the stimulus [15], [16], [17]. Target blanking results in an initial decrease of eye velocity after target disappearance, but internally generated residual pursuit derived from extraretinal mechanisms can keep pursuit eye velocity at a level of about 30% of the preblank pursuit velocity [17].

Previous fMRI studies that have evaluated pursuit performance in patients with schizophrenia have ignored the impact of target velocity on visual motion processing and sensorimotor transformations by testing only one target speed. Therefore, it was our specific intend to use a range of four different target speeds to further unravel the neuronal underpinnings of pursuit eye tracking in schizophrenia. We designed a step ramp paradigm [18] to reduce the frequency of compensatory catch-up saccades during pursuit initiation. Also, target velocity smoothly decreased towards the end of ramps to avoid abrupt direction reversals similar to sinoids or oscillating targets. To evaluate extraretinal components of pursuit generation, intervals with target blanking were used in 50% of trials.

Based on our previous findings, we expected that first, the correlation between target velocity on the one hand and V5 activation and its parietal projection fields on the other hand would be reduced in patients reflecting altered visual motion processing. Second, we expected to observe a neural network of increased activity providing extraretinal information for compensatory mechanisms in patients compared to controls.

## Materials and Methods

### Subjects and Instruction

Sixteen healthy subjects (mean age 27.6 years, SD: 2.5) were matched to seventeen patients with schizophrenia (mean age 29.6 years, SD: 7.1) according to DSM-IV [19]. Patients were in and out patients of the Departments of Psychiatry and Psychotherapy of the Universities Luebeck and Hamburg. Mean duration of illness was 9 years (SD: 6.3, range: 0.5–21). The Positive and Negative Syndrome Scale (PANSS) revealed a mean of 14.8 (SD:7) for positive symptoms, a mean of 20.1 (SD: 8.6) for negative symptoms and a mean of 30.3 for global symptoms [20]. Patients were treated with either amisulpride (N = 5), quetiapine (N = 7), olanzapine (N = 4), ziprasidone (N = 2), aripiprazole (N = 1) or flupentixol (N = 1). Three patients received combinations of antipsychotic medications (two patients on olanzapine + quetiapine and one patient on olanzapine + amisulpride). Mean chlorpromazine equivalents were 393 (SD: 276). All subjects were right handed and had normal vision. Exclusion criteria were any neurological disease or substance abuse. The study was approved by the Local Ethics Committee and all subjects gave informed written consent to participate in the study.

### Experimental Design and Eye Movement Assessment

For this event related fMRI study we used a 2×2×4 design including the between subject factor *group* (patients and healthy subjects) and the within subject factors *blanking* (blanking and non-blanking) and *velocity* (5, 10, 15, 20°/s). We used a step ramp paradigm [18] like in our previous study [16]. In short, we presented target ramps comprising a visual angle of +/−20°. The target (size: ½ degree) was a red dot on a black background projected onto a mirror which was mounted on the head coil. The luminance of the target was 5 candela/m^2^ and that of the background was ½ candela/m^2^. The target moved at constant velocities of either 5, 10, 15 and 20°/s. The direction of target movement was always predictable: when a trial had ended at the right eccentricity (+20°), the next ramp would start at that rightward position and would move from there to the left eccentricity (−20°) and vice versa. One session lasted 27.4 minutes and comprised 160 trials (4 velocities×2 directions×20 repetitions). During 50% of trials the target was blanked off for 1s; this blanking interval always started at position +/−10°, respectively. Ramps with or without target blanking were intermingled in randomized order as were ramp velocities.

The paradigm was demonstrated to the subjects prior to the scanning session to assure optimal tracking performance. Subjects were instructed to always follow the target as accurately as possible and to continue smooth pursuit eye movements at the same velocity whenever the target disappeared during blanking intervals.

Eye movements were recorded during scanning sessions by a Limbus tracker (Cambridge Research Systems, 500 Hz).

### Eye Movement Analysis

Eye movements were analyzed using a semi-automatic computer program developed on the basis of MatLab R13 (The MathWorks In., Natick, MA, USA). Artefacts like blinks, and saccades were removed before analyzing SPEM. Saccades were registered if the initial eye velocity exceeded 30°/s, the amplitude was larger than 0.5° and the duration longer 10ms as in previous studies [16], [21], [22].

For each subject and condition, mean eye velocity and gain (eye velocity/target velocity) was calculated in 1000ms intervals starting whenever the target passed the -10° position for rightward or the +10° position for leftward movements. Mixed ANOVAs including the factors *group* (patients and healthy subjects), *blanking* (yes/no) and *velocity* (5, 10, 15, 20°/s) as provided by SPSS^©^ (Ver. 18.0.3 SPSS Inc., Chicago, Ill, USA) were used to analyze group differences. Subsequent post-hoc tests were applied when appropriate. The Greenhouse - Geisser method was used to correct for non-sphericity.

### Image Acquisition

The study was performed on a 3 Tesla magnetic resonance system (TRIO, Siemens AG, Germany) using a standard head coil. T2*- weighted MRI images were acquired with gradient-echo planar image (EPI) sequences (TR 2.62s, TE 30 ms). Measurement lasted 27.4 minutes with a total of 628 volumes comprising 42 slices each of 3 mm and a distance factor of 18% (0.54 mm gap). Slices were oriented parallel to the AC-PC-line. The subjects’ head was positioned in the head coil by foam pads.

### Image Processing and Statistical Analysis

Data analysis was performed by using SPM5 (Wellcome Department of Cognitive Neurology, London, UK). All volumes were realigned to the first volume, spatially normalized [23] to a standard EPI template and finally smoothed using a 8-mm Gaussian kernel [24]. They were then fitted to a general linear model to establish parameter estimates for each subject. The basis function was a box car function as implemented in SPM5.

The analysis of the data commenced at single-subject level by specifying a model including all experimental conditions in separate regressors. The event was set at the position whenever the target passed the -10° position for rightward and the +10° position for leftward target movement. The event-duration was determined at 1000 ms. In addition, we included the six movement–parameters obtained from the realignment procedure. Then we created contrasts that averaged all 8 instances of each condition and subject. These individual contrast images were then compared on the second level (random effects analysis) in an ANOVA design (*group* (healthy subjects and patients)×*blanking* (blanking and non-blanking)×*velocity* (5, 10, 15, 20°/s)). Since we were interested in the differences between the healthy subjects and the patients, we created linear contrasts testing for the main effect of *group* and those interactions including the factor *group* (*group*×*velocity*, *group*×*blanking*, *group*×*velocity*×*blanking*). To test for higher activations in healthy subjects compared to patients (main effect *group*) contrast codes were set in *healthy subjects* at 1 for all four velocities from non-blanking and blanking conditions whereas contrast codes in *patients* were all set at -1. The reverse contrast codes were used to test for higher activations in patients compared to controls. To test our hypothesis, that BOLD-response changes increase with increasing velocities more strongly in healthy subjects than controls, contrast codes for the interaction *group*×*velocity* in *healthy subjects* were set at 1 for 5°/s, 2 for 10°/s, 3 for 15°/s and 4 for 20°/s target velocities in both non-blanking and blanking conditions, whereas in *patients* contrast codes were set at -1 for 5°/s, -2 for 10°/s, -3 for 15°/s, and -4 for 20°/s target velocities in both non-blanking and blanking conditions. To test the hypothesis, that the more demanding *blanking* task might lead to increased activation we set contrast codes for the main effect ‘blanking’ at -1 for non-blanking and 1 for blanking in the healthy subjects and patients. For the interaction *group x blanking* contrast codes were set at -1 in the healthy subjects for the non-blanking condition and 1 for the blanking condition. In the patient group contrast codes for the non-blanking condition were set at 1 and at -1 for the blanking condition. The conservative threshold for the ANOVA was set at p = 0.05 (Family wise error correction, FWE).

To more specifically determine the relation of target velocity and BOLD-responses we calculated a regression analysis using the least square method in both groups separately. The preprocessing including realignment, normalization and sphericity correction was identical to the ANOVA procedure described above. Target velocities were convolved as delta functions with a canonical hemodynamic response function (HRF) as implemented in SPM5 (parametric modulation). The effects were tested with the appropriate linear contrasts for each condition (blanking and non – blanking). Then the contrast images of the single subjects were analyzed with a one-sample *t*-Test on the second level (random effects analysis). The data were plotted using the coordinates of the Montreal Neurological Institute (MNI). To identify specific eye movement related regions like the frontal *eye* field (FEF) and the supplemental *eye* field (SEF) we used coordinates of previous studies [25], [26], [27], [28]. For all clusters of the oculomotor network of the regression analysis depicted in table 1 we used values from the second level analysis (threshold p = 0.001) and applied small volume correction (10 mm radius spheres) and FWE (Family wise error) correction.

**Table 1:** Regression analysis of target velocity and BOLD.

	*Condition A*					*Condition B*				
upper coordinates: healthycontrols lower coordinates :patients	x	y	z	t-value	P (FWE)	x	y	z	t-value	P (FWE)
Calcarine fissure right	12	−72	−3	11.63	<0.0001	9	−66	3	12.5	<0.0001
	12	−78	6	6.6	<0.0001	18	−60	0	5.74	0.002
Calcarine fissure left	−12	−66	3	9.68	<0.0001	−12	−72	6	16.23	<0.0001
	−3	−78	6	6.53	<0.0001	−21	−57	3	6.96	<0.0001
V5 (hMT/hMST) right	45	−63	3	7.47	<0.0001	51	−63	0	9.16	<0.0001
	42	−63	3	4.27	0.029	48	−60	9	5.65	0.002
V5 (hMT/hMST) left	−39	−72	3	4.86	0.008	−48	−69	6	7.45	<0.0001
	−51	−72	0	3.61	0.082	−39	−78	0	5.90	0.001
Intraparietal sulcus right(putative LIP)	21	−54	51	5.48	0.004	21	−63	57	8.78	<0.0001
	30	−57	57	3.97	0.047	18	−63	51	3.67	0.063
Intraparietal sulcus left(putative LIP)	−24	−60	63	5.03	0.009	−12	−66	54	6.45	0.001
	−27	−57	60	3.89	0.053	−21	−51	51	4.01	0.037
Superior temporal gyrus right	−	−	−	−	−	66	−39	18	4.81	0.013
	−	−	−	−	−	−	−	−	−	−
Superior temporal gyrus left	−	−	−	−	−	−63	−45	15	5.43	0.004
	−	−	−	−	−	−54	−39	21	4.58	0.014
Frontal eye field right	42	−3	54	5.14	0.007	54	6	39	6.77	<0.0001
	45	0	54	4.87	0.008	57	6	42	6.56	<0.0001
Frontal eye field left	−39	−6	51	5.22	0.006	−45	0	48	6.13	<0.0001
	−51	−3	48	5.82	0.001	−54	6	33	5.94	0.001
Supplementary eye field (SEF)right	0	−3	63	4.29	0.026	3	6	54	5.74	0.003
	−	−	−	−	−	−9	15	45	4.24	0.025
Supplementary eye field (SEF)left	0	−3	63	4.29	0.026	3	6	54	5.74	0.003
	−	−	−	−	−	−9	15	45	4.24	0.025
Putamen right	24	9	3	4.24	0.017	21	15	−9	6.15	0.001
	−	−	−	−	−	−	−	−	−	−
Putamen left	−21	6	0	4.39	0.023	−9	12	6	6.64	<0.0001
	−	−	−	−	−		−	−	−	−
Cerebellum Vermis (X)	−	−	−	−	−	3	−57	−39	6.38	0.001
	−	−	−	−	−	−	−	−	−	−
Cerebellum Vermis (VIII)	−	−	−	−	−	−3	−69	−33	4.91	0.013
	−	−	−	−	−	−	−	−	−	−
Cerebellum Vermis (V)patients:(VI)	9	−69	−9	6.08	0.001	−	−	−	−	−
	6	−72	−18	7.98	<0.0001	−	−	−	−	−
Cerebellum Flocculus right (X)	−	−	−	−	−	21	−39	−51	6.45	<0.001
	−	−	−	−	−	−	−	−	−	−
Cerebellum Flocculus left (X)	−	−	−	−	−	−21	−39	−48	5.47	0.003
	−	−	−	−	−	−18	−45	−51	3.71	0.059
Cerebellum posterior lobe right (VI)	−	−	−	−	−	12	−72	−21	8.92	<0.001
	−	−	−	−	−	−	−	−	−	−
Cerebellum posterior lobe left (VI)	−	−	−	−	−	−12	−75	−18	5.51	<0.001
	−	−	−	−	−	−	−	−	−	−
Cerebellum anterior lobe right (V)	−	−	−	−	−	30	−54	−36	5.25	0.007
	−	−	−	−	−	−	−	−	−	−
Cerebellum anterior lobe left (V)	−	−	−	−	−	−21	−42	−30	3.68	0.062
	−	−	−	−	−	−	−	−	−	−

The table shows the results of regression analysis of the velocity on the BOLD signal (Blood Oxygen Level Dependency) in patients and healthy subjects. The significance threshold was set at T = 3.5, family wise error correction (FWE) and small volume correction (SVC) of 10 mm sphere, t-values and P-values are depicted, and x, y and z (mm) refer to the coordinates defined by the Montreal Neurological Institute (MNI).

## Results

### Imaging Data, ANOVA

**Figure 1:**
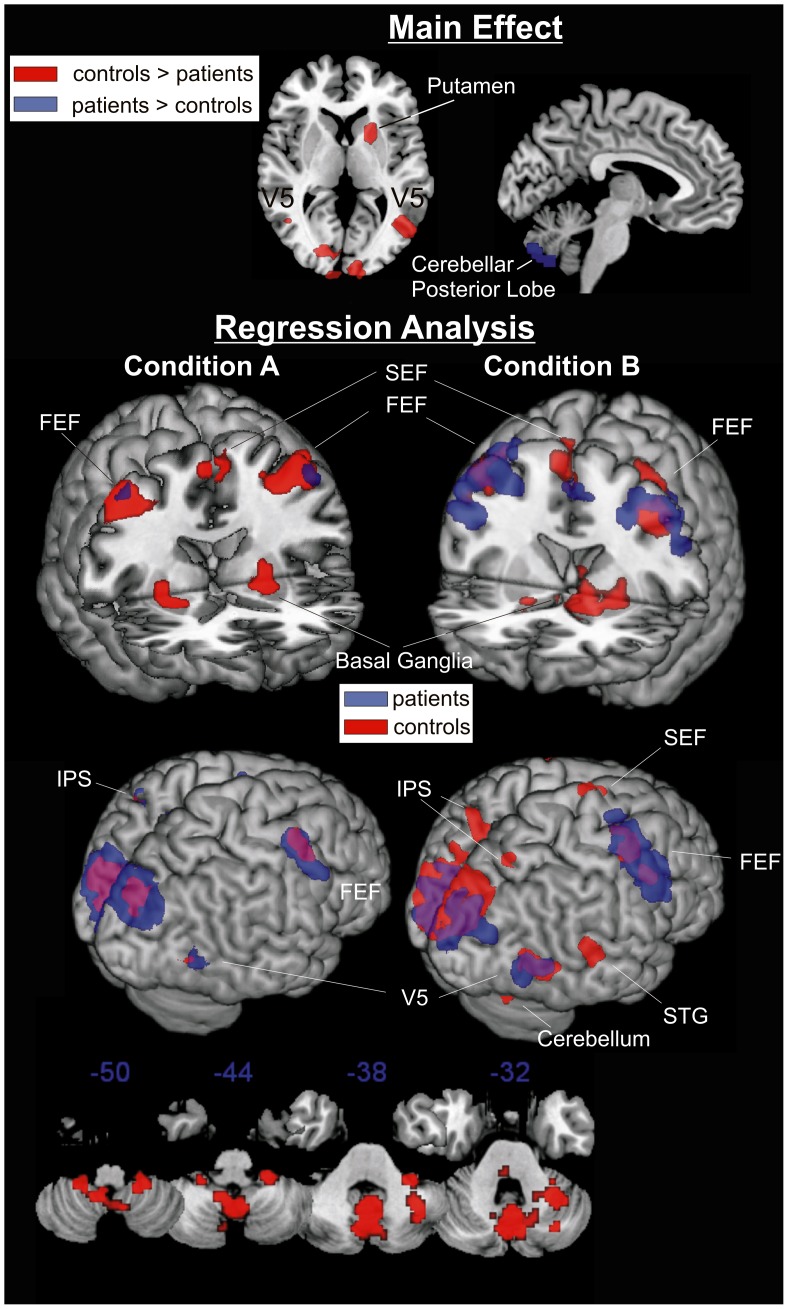
Activated regions revealed by the ANOVA and the regression analysis. Red or blue blobs in the ANOVA represent the calculated main effect (patients > controls and vice versa). For both contrasts family wise error correction at the p = 0.05 level was applied; Condition A: continuous target presentation, Condition B: target blanking. Bottom: Exemplarily the cerebellum during condition B is depicted.

The main effect *group* (controls >patients) revealed that activation of V5 bilaterally and right putamen was higher in healthy subjects than in patients whereas the reverse contrast (patients > controls) showed that in patients activation within cerebellar vermis was higher than in healthy participants (Figure 1, Table 2). For both, the activation in V5 complex and in putamen there was a strong interaction of *group* x *velocity* indicating that activation in these regions increased with increasing target velocity in healthy participants but not in patients. All other interactions which included the factor *group* (*group x blanking* or *group x blanking x velocity*) were not significant.

**Table 2:** Significant main effects and interactions of the ANOVA.

main effect *group* (healthy >patients)	x	y	z	t-value	P (FWE)
V5 right	48	−63	3	9.29	<0.001
V5 left	−45	−60	3	5.04	0.018
Putamen right	21	12	3	6.44	<0.001
**main effect ** ***group*** ** (patients > healthy)**					
Vermis (Uvula)	0	−72	−42	5.96	<0.0001
**interaction ** ***group*** ** x ** ***velocity***					
V5 right	48	−63	3	8.22	<0.001
V5 left	−45	−60	3	4.53	0.127
Putamen right	21	9	3	6.37	<0.001

The table shows the main effect of *group* and the interaction of *group x velocity* as revealed by ANOVA. The significance threshold was set at T = 4.78 family wise error correction (FWE), with one exception: activation difference (interaction *group x* velocity) in V5 left felt just below that threshold. P and t-values are depicted, and x, y and z (mm) refer to the coordinates defined by the Montreal Neurological Institute (MNI).

### Regression Analysis

In conditions with continuous target presentation (non-blanking) target velocity was correlated with activation in the network for pursuit control involving FEF, cerebellar vermis and V1 in both patients and healthy participants. However, activations in supplementary eye field, putamen and cerebellum were not correlated to target velocity in patients in contrast to healthy participants. Activation of V5 and in intraparietal sulcus (putative LIP) bilaterally was also less strongly correlated to target velocity in patients than healthy participants (Figure 1, Table 1).

Target blanking led to a rather increase of correlations between target velocity and cortical activation including the FEF, the SEF and the V5. For some regions such as the superior temporal gyrus and cerebellar regions significant correlations were only observed in conditions with target blanking. Differences between groups were small such as for intraparietal sulcus (putative LIP), SEF, superior temporal gyrus and cerebellar regions (Figure 1).

### Behavioral Data

Analyses of eye movement recordings showed that eye velocity gain was significantly lower during target blanking intervals (condition B) as compared to continuous target presentation (condition A, F_condition_(1, 27) = 103.8, p<0.001). Furthermore, eye velocity gain decreased with increasing target velocity during blanking conditions but not so during non-blanking conditions (F_condition×target velocity_ (3, 25) = 3.95, p = 0.025). Although patients demonstrated lower gain values, there were no statistical significant group differences or interactions indicating unimpaired performance in patients (Table 3).

**Table 3:** Smooth pursuit eye movement (SPEM): velocity and gain.

SPEM VelocitySPEM Gain	Patients		Controls	
	SPEM Velocity	SPEM Gain	SPEM Velocity	SPEM Gain
**Condition A (non-blanking)**				
**5°/s**	4.30 (1.05)	0.86 (0.21)	4.39 (1.80)	0.88 (0.36)
**10°/s**	8.86 (2.32)	0.89 (0.23)	9.47 (2.06)	0.95 (0.21)
**15°/s**	13.48 (3.81)	0.90 (0.24)	13.85 (3.60)	0.92 (0.24)
**20°/s**	17.79 (3.11)	0.89 (0.17)	18.38 (5.06)	0.92 (0.25)
**Condition B (blanking)**				
**5°/s**	3.70 (1.64)	0.74 (0.33)	4.17 (0.93)	0.83 (0.19)
**10°/s**	6.62 (2.38)	0.66 (0.24)	6.86 (2.35)	0.69 (0.23)
**15°/s**	9.90 (3.81)	0.66 (0.25)	10.65 (4.11)	0.71 (0.27)
**20°/s**	11.50 (3.11)	0.58 (0.16)	12.58 (5.34)	0.63 (0.27)

Mean smooth pursuit eye movement (SPEM) – gain values and corresponding velocities and saccadic frequencies with standard deviations during the event interval of 1000 ms in conditions A and B. There were no significant differences between the patients and the healthy subjects for any parameter.

## Discussion

The aim of this study was to evaluate velocity processing during active pursuit of target ramps in patients with schizophrenia by using for the first time a range of four different target velocities in an event related fMRI design. The eye movement data from scanning sessions indicate that patients’ pursuit performance was not impaired compared to controls, neither in non-blanking nor blanking conditions. Thus, activation differences between groups were independent of possible alterations in performance. This observation replicates earlier findings from fMRI studies that showed alterations in the neural network for pursuit in patients despite unimpaired pursuit performance [6], [28], [29].

Our main findings indicate reduced neuronal activation in motion perception area V5 bilaterally and in right putamen in patients compared to healthy participants. This group difference was modulated by target velocity as revealed by significant interactions between *group* x *velocity* in ANOVA. More detailed regression analysis for continuous visual pursuit (non-blanking conditions) showed that neural activation in V5 was less strongly correlated with target velocity in patients than controls. Activation in neither putamen bilaterally nor SEF was correlated with target velocity in patients in contrast to healthy participants. Furthermore, patients revealed stronger activation of the cerebellar vermis than healthy participants. However, this cerebellar activation was not correlated with target velocity in patients, suggesting that this possibly increased compensatory activity was not directly linked to target velocity. Together, these findings imply that velocity processing in an occipito-parieto-frontal network for pursuit control is impaired in patients with schizophrenia.

Removing the visual target during ongoing pursuit resulted in a decrease of eye velocity in both groups similarly. In line with this, target blanking had only little effects on activation differences between groups, e.g. no interaction of *group x target blanking* were observed in ANOVA.

In all, the results of this study suggest that velocity processing is impaired in patients with schizophrenia and that patients recruit different extraretinal mechanisms than healthy individuals to compensate for this deficit.

### Dysfunction of Visual Motion Processing in V5 and Parietal Areas

Sensory processing deficits in schizophrenia have been attributed to both: a deficiency of motion detection as well as impaired visual perception [3], [30], [31], [32]. In line with this model, which has been concluded from psychophysiological experiments [3], [31], [33], [34] we found decreased velocity related activation in V5 and in the intraparietal sulcus in patients compared to controls. This finding supports the hypothesis of impaired feed forward transfer of motion information along the dorsal stream of the magnocellular pathway from V5 to parietal association cortex [29]. Functionally, the magnocelullar pathway involves eye movement control, action guidance, initial attention modulation, motion perception, and visual/somatosensory integration [35]. The present finding further underlines earlier studies that showed a reduced correlation between the measured eye velocity and the BOLD response in V5 probably reflect a deficient internal representation of target velocity that can not be adequately used for eye movement control in patients [21], [36]. Butler and Javitt [30] reported reduced visual evoked potentials in patients during presentation of low luminance contrast stimuli which specifically stimulate the magnocellular system. Our moving stimulus was a red dot on a black background with a low contrast suggesting intense magnocellular involvement leading to reduced activation of V5 area in patients.

### The Role of the Supplementary Eye Field

The SEF has been found to be related to prediction, oculomotor learning, assessment of motion direction, generation of the oculomotor command and the coding of the eye velocity signal [27], [37], [38], [39], [40], [41], [42]. In the present study, we found no general group difference for SEF activation but regression analyses showed a lack of correlation between target velocity and the upper SEF in patients during pursuit of continuously visible targets. This finding is in line with an earlier finding that showed reduced SEF activation during pursuit in patients that were matched for pursuit performance with healthy controls [13], [28]. It implies that a sensorimotor transformation deficit in SEF with visible targets contributes to the SPEM deficit in patients with schizophrenia.

Otherwise, during target blanking patients were able to enhance velocity related SEF activation, presumably by extraretinal input. However, activation during target blanking was located lower in the SEF in patients (z = 45) than healthy subjects (z = 54), suggesting that patients rather use subregions of the SEF which are related to memory as a part of prediction of target motion [38].

### Basal Ganglia (Striatum)

There is strong evidence that neural activity in networks that interconnect different cortical areas is modulated by feedback information via the basal ganglia and the cerebellum [43], [44]. Neggers et al. [45] found saccade related activation in the putamen. Accordingly, we have recently demonstrated reduced correlation of motion processing related activity in V5 and activation of the basal ganglia, i.e. caudate, in patients with schizophrenia compared to controls [29]. This finding implies that during pursuit visual motion information for eye tracking control is less available in basal ganglia in patients than controls. In the present study, ANOVA and regression analysis revealed a lack of activation in the basal ganglia, i.e. the putamen, in patients compared to controls during both continuous target presentation and target blanking. Such a deficit which is independent of the presence or absence of the target, suggests a motor learning related deficit. The Striatum has also been found to be related to reward and motivation [46]. The execution of an accurate movement could be the reward that minimizes both error and effort terms in overall cost [47].

### Cerebellum

Activation of the cerebellar vermis (uvula) was increased in the patient group in the ANOVA when compared to the healthy subjects. Increased activation of the vermal uvula in patients as revealed by ANOVA seems to represent compensatory activation, since the uvula has been found to be related to ‘compensation for the visual consequences of pursuit’ as Krauzlis et al. reported [48], [49], [50]. However, regression analysis revealed that this increased cerebellar activation was considerably less strongly correlated to target velocity in patients than controls (see Figure 1).

The final path of the SPEM signal presumably passes trough the cerebellum as ablation of the cerebellum seriously impairs SPEM [51], [52]. The oculomotor vermis is a major end point of the corticopontocerebellar pathway underlying the translation of target motion into a premotor pursuit command, and vermal purkinje cells are related to the velocity and the dynamics of SPEM [48], [52], [53], [54].

We found a brought network of affected regions in the patients supporting the hypothesis of a deficient interaction between different regions. Andreasen and Pierson [55] who introduced the concept of ‘cognitive dysmetria’ in schizophrenia which is caused by the cerebellum also stated, that ‘the role of the cerebellum is probably not primary in the sense that is the sole region that is dysfunctional’. Since we found a lack of velocity related activation of the vermis in our study, we suggest now and more specific a deficient transmission of the signal of target and eye-movement velocity. Whether the signal is stronger related to the eye movement component [56] or the oculomotor signal to guide the eye movements remains speculative.

Increased activation of the vermal uvula during the ANOVA seems to represent compensatory activation, since the uvula has been found to be related to ‘compensation for the visual consequences of pursuit’ as Krauzlis reported [48], [49], [50].

### Limitations

We minimized saccadic frequency during pursuit by using a step ramp paradigm in which velocity smoothly decreased towards the turning point at the end of ramps and by giving clear instructions to the subjects to always continue eye tracking even when the target was not visible. However, we cannot exclude the influence of saccades on activations, in particular during condition B. We did not find group differences for this blanking induced increase of saccades in the eye movement data, neither was there a significant interaction of group x blanking in the fMRI data. It thus appears unlikely to us that group differences in activation could be due to saccadic related activity.

Given the event related study design, the integration of all measured eye movement data for each ramp/event would have been optimal. In fact, just ∼70% of the behavioral data were available for quantitative analyses. Since extrapolation of the missing data would have been too imprecise we decided not to use the behavioral data in the SPEM model but rather use target velocity in regression analysis. Behavioral data of the otherwise sufficient SPEM performance supports our hypothesis that modulating the BOLD-response with the presented target velocity represents a valid method to analyze velocity coding of SPEM.

### Conclusions

In conclusion, our results support a model of altered velocity processing in a neural network for pursuit control that involves mechanisms of perception as well as retinal and extraretinal velocity processing in patients with schizophrenia. The findings suggest deficiencies on different levels of target velocity processing and perception but not a single region. On a more general level, our results give a new insight into the neurophysiological mechanisms of motion perception and its use for action control, i.e. oculomotor control, in schizophrenia.
